# *O*^*6*^-*methylguanine-DNA methyltransferase *is downregulated in transformed astrocyte cells: implications for anti-glioma therapies

**DOI:** 10.1186/1476-4598-6-36

**Published:** 2007-06-05

**Authors:** Ken Sasai, Tsuyoshi Akagi, Eiko Aoyanagi, Kouichi Tabu, Sadao Kaneko, Shinya Tanaka

**Affiliations:** 1 Laboratory of Molecular and Cellular Pathology, Hokkaido University Graduate School of Medicine, W15 N7, Kita-ku, Sapporo 060-8638, Japan; 2 KAN Research Institute Inc., 6-7-3 Minatojima-minamimachi, Chuo-ku, Kobe 650-0047, Japan; 3 Kashiwaba Neurosurgical Hospital, 15-7-20, Tsukisamu E1, Toyohira-ku, Sapporo 062-8513, Japan

## Abstract

**Background:**

A novel alkylating agent, temozolomide, has proven efficacious in the treatment of malignant gliomas. However, expression of *O*^*6*^*-methylguanine-DNA methyltransferase *(*MGMT*) renders glioma cells resistant to the treatment, indicating that identification of mechanisms underlying the gene regulation of *MGMT *is highly required. Although glioma-derived cell lines have been widely employed to understand such mechanisms, those models harbor numerous unidentified genetic lesions specific for individual cell lines, which complicates the study of specific molecules and pathways.

**Results:**

We established glioma models by transforming normal human astrocyte cells via retroviral-mediated gene transfer of defined genetic elements and found that MGMT was downregulated in the transformed cells. Interestingly, inhibitors of DNA methylation and histone deacetylation failed to increase MGMT protein levels in the transformed astrocyte cells as well as cultured glioblastoma cell lines, whereas the treatment partially restored mRNA levels. These observations suggest that downregulation of MGMT may depend largely on cellular factors other than promoter-hypermethylation of *MGMT *genes, which is being used in the clinic to nominate patients for temozolomide treatment. Furthermore, we discovered that Valproic acid, one of histone deacetylase inhibitors, suppressed growth of the transformed astrocyte cells without increasing MGMT protein, suggesting that such epigenetic compounds may be used to some types of gliomas in combination with alkylating agents.

**Conclusion:**

Normal human astrocyte cells allow us to generate experimental models of human gliomas by direct manipulation with defined genetic elements, in contrast to tumor-derived cell lines which harbor numerous unknown genetic abnormalities. Thus, we propose that the study using the transformed astrocyte cells would be useful for identifying the mechanisms underlying MGMT regulation in tumor and for the development of rational drug combination in glioma therapies.

## Background

Gliomas, accounting for 30% of adult primary brain tumors, are the most common primary tumors of the central nervous system and are classified into four clinical grades, with the most aggressive and lethal tumors being grade IV glioblastoma multiforme (GBM) [1,2]. The median survival of GBM patients is less than one year from initial diagnosis, and many of the commonly used chemotherapeutic agents have limited effects on these malignant tumors [3]. Recently, there has been increasing hope that temozolomide, a novel alkylating agent, will prove efficacious in the treatment of human glioma [4,5]. However, a number of studies have suggested that, in tumors, *O*^*6*^-methylguanine-DNA methyltransferase (MGMT) provides resistance to treatment with temozolomide, unless expression is lost by promoter methylation or there is direct inhibition of MGMT activity [6]. Considering the attractive efficacy of temozolomide, one of the greatest challenges facing the field may be to identify therapeutic agents that suppress *MGMT *expression, as such drugs may sensitize resistant glioma cells to temozolomide. Thus, establishment of more sophisticated systems to understand the functions and regulation of MGMT are highly desired.

Normal human cells, genetically modified by retroviral-mediated gene transfer, have proven important, because such systems are useful for identifying factors directly contributing to tumorigenesis, in contrast to tumor-derived cell lines which harbor numerous unknown genetic abnormalities [7,8]. GBMs are believed to arise from astrocyte cells by means of stepwise accumulation of genetic abnormalities [4,9], and immortalized normal human astrocyte (NHA) cells had been established by introducing the telomerase catalytic subunit (hTERT) in combination with human papillomavirus E6/E7 to inactivate both p53 and pRb pathways [10]. It had been systematically demonstrated that the immortalized NHA cells, expressing activated Ras (H-RasV12) or expressing both H-RasV12 and an active form of Akt (myrAKT), formed tumors consistent with human anaplastic astrocytoma or GBM in intracranial- and flank-xenografts models [10,11]. These studies indicate that such systems are useful for glioma research to understand direct functions and regulation of genetic elements during transformation and gliomagenesis.

Here we created similar experimental models using NHA cells by introducing the simian virus 40 early region (*SV40ER*) instead of human papillomavirus E6/E7. The *SV40ER *encodes both small-t antigen, a suppressor of protein phosphatase 2A (which is downregulated in half of human glioma) [12] and large-T antigen, which directly binds to and inactivates p53, as well as pRB and the closely related proteins p107 and p130 [13]. Using such genetically modified NHA cells, we demonstrate that MGMT is downregulated during oncogene-mediated transformation of astrocyte cells. Since our results indicate that downregulation of MGMT expression was not primarily dependent upon promoter hypermethylation, inhibitors of DNA methylation or histone deacetylases (HDACs) may be used in combination with alkylating agents for improved treatment of some GBM cases. We propose that the NHA cell systems are useful for investigating the mechanisms underlying MGMT expression and for improving glioma therapies.

## Results

**Figure 1:**
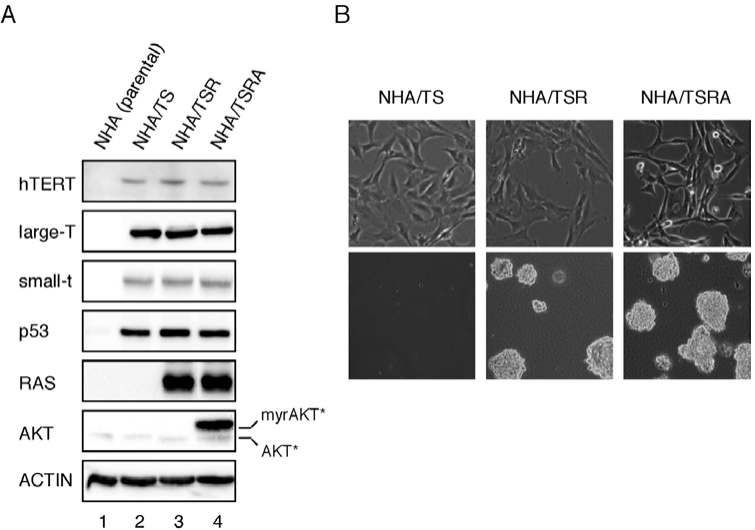
**Establishment of immortalized and transformed NHA cells**. (A) Protein extracts (10 μg) from NHA cells infected with retroviral vectors expressing indicated genes (T, hTERT: S, SV40ER: R, H-RasV12; A; myrAKT) were analyzed by immunoblotting. Asterisk, active form of AKT (myrAKT) was distinguishable from wild-type form. (B) The morphologies (top panels) and anchorage-independent growth properties (bottom panels) of NHA/TS, NHA/TSR, and NHA/TSRA cells are shown.

**Figure 2:**
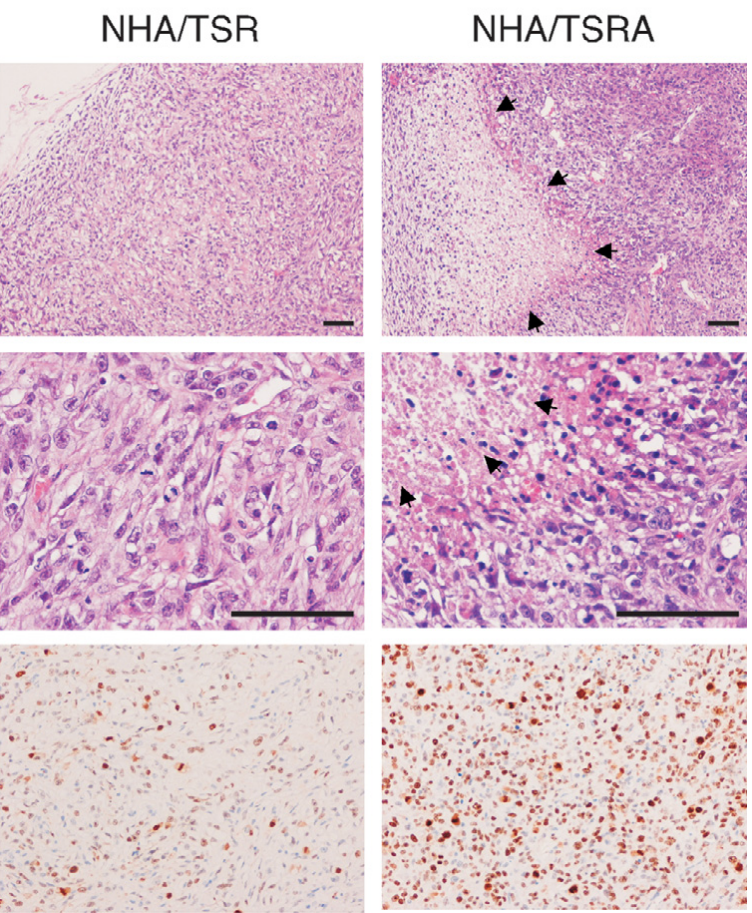
**Histological features of s.c. xenografts derived from the transformed NHA cells**. Formalin-fixed paraffin-embedded tissue sections (5-μm-thick) of xenografts derived from NHA/TSR (left) and NHA/TSRA (right) cells were subjected to histological analyses. Low- (top) and high- (middle) magnification images (H&E staining; scale bar, 200 μm) and immunohistochemistry using an anti-Ki-67 antibody (bottom) are shown.

To create an experimental model of human gliomas, NHA cells were introduced with genes for *hTERT *(T), *SV40ER *(S), *H-RasV12 *(R), and *myrAKT *(A) (Figure 1A; NHA/TS, NHA/TSR, NHA/TSRA cells). The NHA/TS cells grew much faster than parental NHA cells, exhibiting continuous growth beyond population-doubling 50 (data not shown). However, the NHA/TS cells did not form colonies in soft agar or tumors in xenografts into recipient nude mice (Figure 1B; Table 1), indicating that the NHA/TS cells were immortalized but not transformed. In contrast, the NHA/TSR and NHA/TSRA cells formed a considerable number of colonies in soft-agar, as well as tumors in flank-xenografts into nude mice (Table 1). NHA/TSRA cells displayed much more refractile morphology and loose attachment to the culture dish relative to NHA/TSR cells (Figure 1B). Although the difference in the anchorage-independent growth property was quite subtle between NHA/TSR and NHA/TSRA cells (Table 1), the histopathological features of two types of flank xenografts were clearly different. In tumor tissues formed by NHA/TSR cell injection, atypical astrocytic cells with higher cellularity exhibits histopathological features of Grade III glioma as anaplastic astrocytoma of WHO classification. On the other hand, the TSRA-xenografts composed of similar atypical astrocytes displayed numerous areas of necrosis with nuclear pseudo-palisading pattern. Consistent with the histological feature as GBM, the immunohistochemical index of Ki-67 positivity is higher in NHA/TSRA tumors than those of NHA/TSR derived tumors (Figure 2).

**Table 1:** Summary of soft agar colony formation assay and xenograft propagation experiment: NHA and NIH3T3 cells infected with retroviral vectors expressing hTERT (T), SV40ER (S), H-RasV12 (R), myrAKT (A), and/or MGMT as well as parental NHA cells were subjected to the soft-agar colony formation and xenograft propagation assays.

*Cell types*	*Colony numbers *^*a*^	*% Tumor incidence (n) *^*b*^
NHA (parental)	0	ND
NHA/TS	0	0 (3)
NHA/TSR	664 ± 19	100 (3)
NHA/TSRA	736 ± 51	100 (3)
NHA/TSR + MGMT	649 ± 34	ND
NIH3T3/R	1440 ± 56	ND
NIH3T3/R + MGMT	1344 ± 88	ND

^a^NHA (2 × 10^4^) and NIH3T3 cells (1 × 10^4^) were plated in soft-agar (0.36% top agar containing 5% fetal calf serum in 60-mm dishes) and incubated for 21 days and 14 days, respectively. Colonies were stained with 3-(4,5-dimethylthiazol-2-yl)-2,5-diphenyltetrazolium bromide, and the numbers of stained colonies were counted. Results represent mean ± SD from two independent triplicate experiments.

^b^3 × 10^6 ^cells were injected s.c. into nude mice. *n*, number of animals treated. ND, not done.

**Figure 3:**
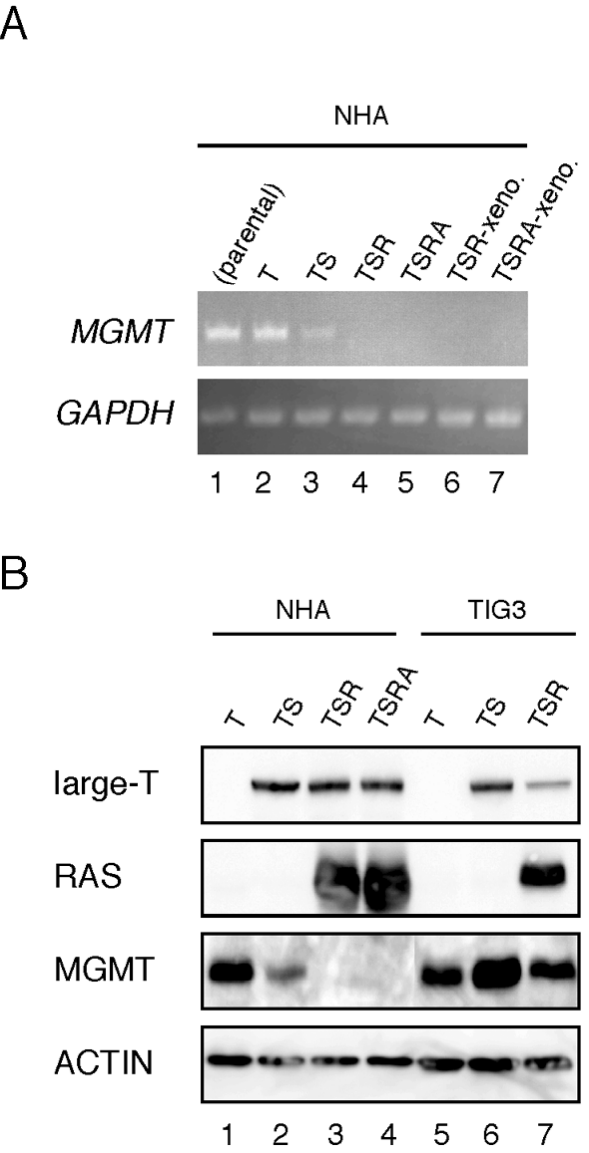
**MGMT is downregulated in immortalized and transformed NHA cells**. (A) The expression of *MGMT *mRNA in NHA (parental; lane 1), NHA/T (lane 2), NHA/TS (lane 3), NHA/TSR (lane 4), and NHA/TSRA cells (lane 5) were analyzed by semi-quantitative RT-PCR. The levels in flank xenografts derived from the NHA/TSR and NHA/TSRA cells were also tested (lanes 6 and 7). (B) Protein extracts (10 μg) from NHA (lanes 1–4) and TIG3 cells (lanes 5–7) infected with retroviral vectors expressing indicated genes (T, hTERT: S, SV40ER: R, H-RasV12; A; myrAKT) were analyzed by immunoblotting.

Using the NHA cell system described above, we found that the mRNA level of *MGMT *was decreased in immortalized (NHA/TS) and transformed (NHA/TSR and NHA/TSRA) cells and that *MGMT *expression was also downregulated in the flank xenografts derived from NHA/TSR and NHA/TSRA cells (Figure 3A). Immunoblot analyses also demonstrated that introduction of *SV40ER *decreased MGMT level in NHA cells and that the levels were further decreased in transformed NHA cells (Figure 3B). Conversely, in human lung fibroblasts TIG3/T cells (TIG3 cells expressing hTERT) [14], neither *SV40ER *nor *H-RasV12 *suppressed MGMT expression (Figure 3B), suggesting the astrocyte-specific mechanisms of MGMT regulation. This is an interesting observation as TIG3/TS cells are refractory to Ras-induced genetic alteration [15,16], morphological changes [17] and thereby cellular transformation [14]. Although such observation prompted us to further test the tumor suppressive functions of MGMT, the additional expression of MGMT in NHA/TSR and NIH3T3/R cells (NIH3T3 cells transformed by H-RasV12) failed to suppress anchorage-independent growth (Table 1), suggesting that MGMT may be unable to restore transformed phenotypes. In fact, tumors have long been noted to be heterogeneous in *MGMT *expression [5,6], and approximately half of malignant gliomas expressed MGMT [18]. However, it is possible that the mechanisms underlying resistance to downregulation of MGMT may render TIG3 fibroblasts refractory with respect to oncogene-mediated transformation, because MGMT functions to protect normal cells from exogenous carcinogens [6]. In support of this idea is the observation that *MGMT *mRNA was downregulated in v-fos-transformed [19,20] and Ras-transformed (TA unpublished data) rodent fibroblasts, showing highly malignant phenotypes.

**Figure 4:**
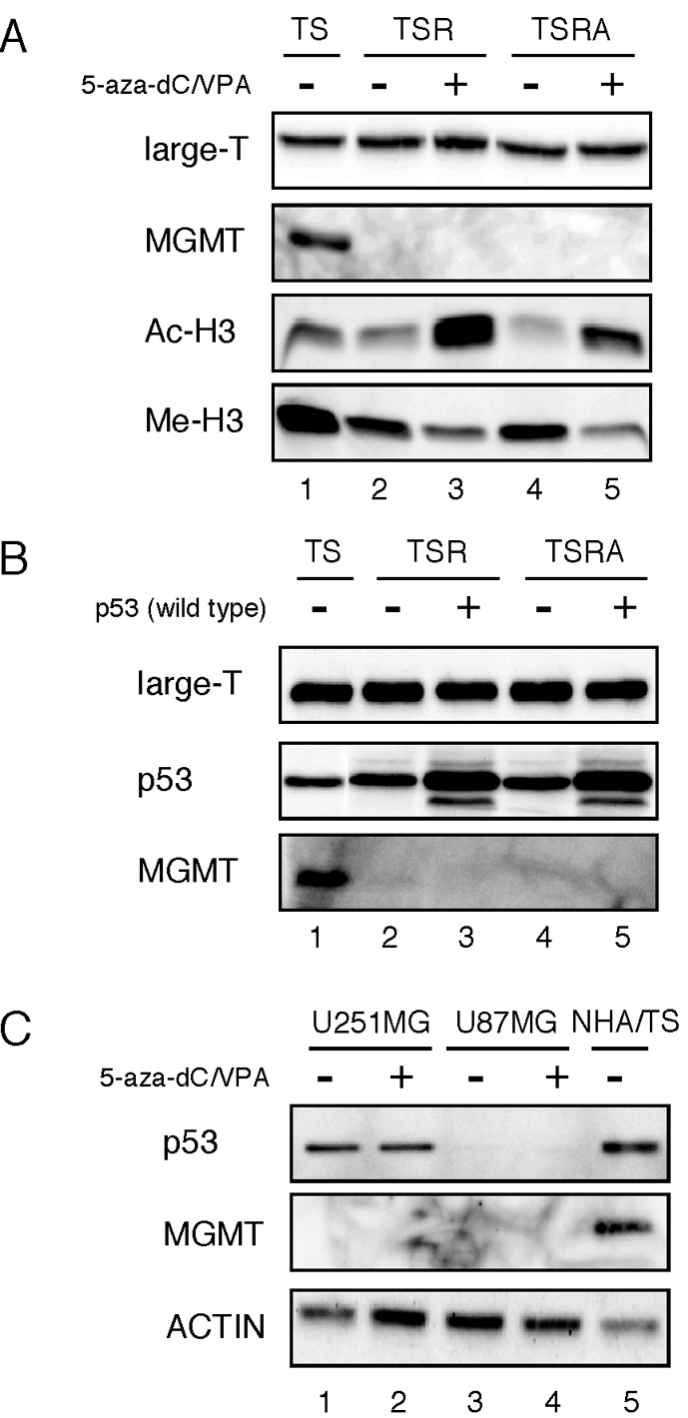
**Neither treatment with 5-aza-dC/VPA nor p53 expression restores MGMT protein levels in transformed NHA cells**. (A) Protein extracts (10 μg) from the transformed NHA cells treated (+) and untreated (-) with 5-aza-dC/VPA were analyzed by immunoblotting. (B) Protein extracts (10 μg) from the transformed NHA cells infected with wild type p53 (+) and control virus (-) were analyzed by immunoblotting. (C) Protein extracts (10 μg) from glioma cell lines treated (+) and untreated (-) with 5-aza-dC/VPA were analyzed by immunoblotting.

To understand the mechanism underlying the downregulation of MGMT in transformed NHA cells, we first analyzed the *MGMT *expression following treatment with 5-aza-2'-deoxycytosine (5-aza-dC) and/or Valproic acid (VPA), because it has been widely acknowledged that aberrant methylation of *MGMT*-promoter contributes to the gene expression changes [6]. Inhibitors of HDACs, such as VPA and trichostatin A, act synergistically with 5-aza-dC (a DNA methyltransferase inhibitor and demethylating agent in dividing cells) to further increase the expression of genes silenced in association with promoter hypermethylation [21]. Reverse transcriptase (RT)-PCR analysis revealed that treatments with 5-aza-dC alone or combined treatment with 5-aza-dC/VPA slightly increased *MGMT *mRNA levels, when the PCR was performed in a condition where the positive control reaction (mRNA from the NHA/TS cells) was saturated (data not shown), suggesting that the downregulation may have been partially mediated by promoter hypermethylation. However, combined treatment with 5-aza-dC/VPA was insufficient to restore MGMT protein levels in NHA/TSR and NHA/TSRA cells whereas the treatment clearly induced acetyltation and demethylation of histone H3 (Figure 4A), suggesting that additional factors, which are expressed in the parental NHA cells but inactivated in the transformed NHA cells, may be required to increase MGMT protein levels.

**Figure 5:**
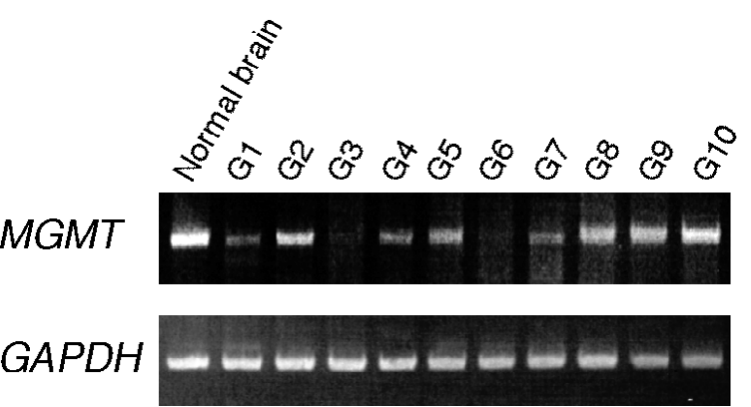
**Expression of *MGMT *RNA in human glioma tissues**. The mRNA levels of MGMT expression in human glioma tissues were analyzed by semi-quantitative RT-PCR.

In an attempt to identify such missing factors contributing to gene regulation of MGMT, we tested if the expression of wild type p53 restores MGMT levels, as MGMT was slightly decreased in the NHA/TS cells where p53 is inactivated by large T antigen (Figure 3). Although several glioma studies have demonstrated correlations between inactivation of the p53 pathway and lower levels and/or activity of MGMT [22-24], the introduction of wild type *p53 *failed to restore MGMT protein levels in transformed NHA cells (Figure 4B). We also confirmed previous reports that both U87MG (harboring wild type *p53*) and U251MG (harboring *p53 *mutations) glioma cells expressed low or no levels of MGMT [25-27]. We further found that treatment with 5-aza-dC/VPA failed to induce MGMT protein expression in both cell lines (Figure 4C), whereas the treatment slightly increased the mRNA levels (by RT-PCR; data not shown). Since gliomas harboring p53 mutations and immuno-positive for p53 (G2, G7, and G8) as well as gliomas harboring wild type p53 and immuno-negative for p53 (G4 and G10) expressed *MGMT *mRNA (Figure 5, Table 2), MGMT might be regulated primarily by the factors other than p53 in gliomas. Conversely, MGMT is clearly downregulated in normal astrocyte cultures from p53 deficient mice [28], suggesting the differences in the regulation of MGMT between normal and transformed cells.

**Table 2:** Diagnosis and p53 status of human gliomas: The index of Ki-67 staining and p53 status (mutation and immunopositivity) are listed with patient ID, age at surgery, gender (F, female; M, male), and clinical diagnosis (GBM, glioblastoma, WHO grade IV; AA, anaplastic astrocytoma, WHO grade III, AE anaplastic ependymoma, WHO grade III). Images of H&E staining and immunohistochemistry are shown [see additional file 1].

*ID*	*Age*	*Gender*	*Diagnosis*	*Ki-67 (%) *^*a*^	*p53 mutations *^*b*^	*p53 staining *^*c*^
G1	73	F	GBM	4.0	N	+
G2	58	M	GBM	44.3	R156P, H214R	++
G3	36	M	GBM	24.8	N	-
G4	77	F	GBM	92.5	N	-
G5	53	M	GBM	12.6	N	++
G6	61	M	GBM	43.6	N	+
G7	83	M	GBM	53.1	H193P	++
G8	28	M	GBM	29.0	H214R	+++
G9	55	F	AA	3.3	N	+++
G10	42	F	AE	42.6	N	-

^a^MIB-1 labeling index (by anti-Ki-67 antibody) was analyzed using the MetaMorphV7.0 software (Molecular Cevices, Downingtown, PA, USA), by counting 500–2,000 nuclei per case.

^b^p53 cDNA was amplified by RT-PCR and the products were directly sequenced. N, no mutation was detected.

^c^Staining intensity was classified into four categories: +++, strong; ++, moderate; +, weak; -, negative.

**Figure 6:**
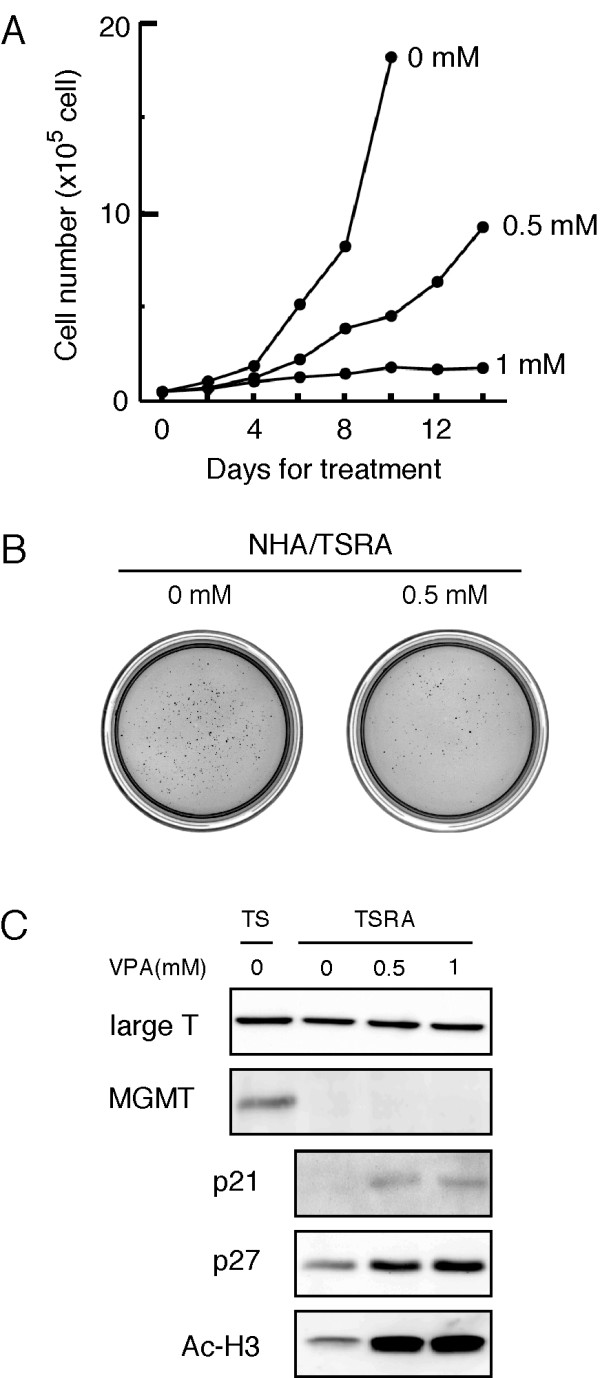
**VPA inhibits cell growth in transformed human astrocyte cells**. (A) Dose-dependent anti-proliferative effects of VPA were shown. NHA/TSRA cells were treated with 0, 0.5 and 1.0 mM VPA for 14 days. Cell numbers were counted every other day and results were expressed in the mean of two independent experiments. (B) NHA/TSRA cells, treated (0.5 mM) and untreated (0 mM) with VPA for two weeks, were subjected to soft-agar colony formation assay. 2 × 10^4 ^cells were plated, incubated at 37°C for 21 days and stained with 3-(4,5-dimethyl-thiazol-2-yl)-2,5-diphenyltetrazolium bromide. Note VPA was not included in the assay media. (C) Protein extracts (10 μg) from NHA/TSRA cells treated with VPA were analyzed by immunoblotting using indicated antibodies.

Since MGMT seems to be regulated in several ways, it is possible that inhibitors of HDACs may be used for therapies for some glioma patients in combination with alkylating agents, such as temozolomide. Since VPA, which inhibits both class I and class II HDACs [29], has displayed potent *in vitro *and *in vivo *antitumor activities against brain tumor cells [30], it is one attractive candidate agent. The treatment with VPA appreciably inhibited growth of the NHA/TSRA cells in a dose dependent manner (Figure 6A) and two-week-treatment with 0.5 mM VPA (within the range of serum levels achieved in pediatric high-grade glioma patients) [31] suppressed anchorage-independent growth (Figure 6B). Although hyperacetylation of histone H3 was noted in the NHA/TSRA cells treated with VPA for two weeks, the treatment failed to restore protein (Figure 6C) and mRNA levels of MGMT (data not shown). Since protein levels of p21^WAF1 ^and p27^KIP1 ^were elevated in VPA-treated NHA/TSRA cells (Figure 6C), the antiproliferative effect might be mediated, at least in part, by cell cycle arrest, as widely acknowledged. These findings suggest that HDAC inhibitors, which suppress tumor cell growth without affecting MGMT expression, may be used in combination with alkylating agents to some glioma patients.

## Discussion

Much of our understanding of the molecular basis of gliomagenesis derives from the study of established cell lines that are explanted from human tumors. Such cell lines are often assumed to be representative of the original diseases, and they have been extensively employed for the identification and preclinical testing of potential therapeutic compounds. However, they harbor an unknown number of genetic lesions, which complicates the study of specific molecules and pathways. Indeed, with respect to p53-mediated MGMT regulation, there have been contradictory observations, where transient knockdown of *p53 *caused MGMT downregulation in SF767 glioma cells without affecting promoter methylation [28] but increased MGMT expression in T98 cells [27]. Since such discrepancy may depend on unidentified mutations specific for individual cell lines, it is difficult to provide constant conclusions using tumor-derived cell lines alone. Therefore, normal human cells as well as genetically engineered mouse models will prove very useful, which allow us to generate experimental models of human cancers by direct manipulation with defined genetic elements [7,8].

Here we established glioma models from NHA cells and demonstrated that MGMT is downregulated in the transformed astrocyte cells. Although numerous studies have proposed the strong linkage between *MGMT *expression and promoter hypermethylation of the gene, treatment with epigenetic compounds (5-aza-dC/VPA) was unable to increase MGMT protein levels in the transformed NHA cells or in cultured tumor cells. Some previous reports demonstrated the epigenetic regulation of *MGMT *expression by treating tumor cell lines with 5-aza-dC and testing the *MGMT *levels by RT-PCR [32,33]. However, based on the data presented here, such limited evaluation might not be sufficient, as RT-PCR assay overestimates the *MGMT *restoration. Since inappropriate evaluation would mislead the development of advanced therapies, protein levels or enzyme activity of MGMT in the cells treated with such compounds should be examined and compared with those in normal cells. In fact, the use of alkylating agents in combination with HDAC inhibitors has been hampered, probably because it has been suspected that such agents would increase MGMT expression. However, we showed that two-week-treatment with VPA inhibited tumor cell growth without increasing MGMT expression, suggesting potential clinical use although further preclinical studies are required.

As the treatment with 5-aza-dC/VPA slightly increased mRNA levels of *MGMT *in cultured glioma cells (including U87MG, U251MG and transformed NHA cells), methylation status might be involved in MGMT regulation to some extent. However, such treatment was insufficient to restore protein expression of MGMT, suggesting that the regulation mechanisms may largely depend on other cellular factors. Although p53 does not seem to contribute directly to MGMT expression, it is possible that other transcription factors, whose expressions have been downregulated or silenced during transformation and gliomagenesis, may collaborate with HDAC inhibitors to increase MGMT protein. Interestingly, the sonic hedgehog pathway has been shown to regulate the self-renewal of CD133-positive glioblastoma cells, which were resistant to temozolomide treatment [34]. Furthermore, some of CD133-positive glioma cultures highly expressed *MGMT *as well as target genes of the sonic hedgehog pathway [35]. Thus, it is possible that the regulation of MGMT expression may be partially mediated by the sonic hedgehog pathway in some cases.

## Conclusion

As discussed above, MGMT seems to be regulated by a number of ways in gliomas. The detailed mechanisms should be further analyzed as MGMT level is a critical determinant for efficacy of therapies with alkylating agents. Thus, identification of molecules and compounds that increase MGMT expression by screening NHA/TSR or NHA/TSRA cells with cDNA- and chemical-libraries would be very useful for the development of rational drug combination. We propose that the NHA cell system creates refined human glioma models for the systematic dissection of genetic alterations and elucidation of the complexities of the signaling pathways important for gliomagenesis. These systems also provide powerful means to find and authenticate molecules of particular promise for therapeutic targeting, and the present study provides an important proof-of-principle test for such systems.

## Methods

### Clinical samples

Brain tumor specimens were obtained, after informed consent, from patients undergoing tumor resection at the Kashiwaba Neurosurgical Hospital.

### Cell culture

NHA cells (Cambrex Bio Science, Walkersville, MD, USA) were cultured in the astrocyte growth medium (AGM; Cambrex Bio Science). All other cells including immortalized NHA cells were maintained in Dulbecco's modified eagle medium (Seikagaku Co., Tokyo, Japan), supplemented with 10% fetal calf serum, 1 mM Glutamine, 50 units/ml penicillin G and 50 μg/ml streptomycin. All cultures were incubated at 37°C under a humidified atmosphere of 95% air and 5% CO2. For the combination treatment (5-aza-dC/VPA), 5-aza-dC (1 μM; Sigma, St. Louis, MO, USA) was added for an initial incubation of 48 h, after which VPA (1 mM; Sigma) was added for an additional 24 h.

### Retroviral vectors and retroviral-mediated gene transfer

A cDNA fragment encoding murine ecotropic retrovirus receptor (*EcoVR*) was obtained from the retroviral plasmid pCX4hyg-EcoVR [14], and then subcloned into pCX4redEx vector [GenBank: AB296084]. Myc-His-tagged active form of mouse *AKT1 *cDNA, which has N-terminal myristoylation, was isolated from the pUSEamp-myr-AKT plasmid (Upstate, Charlottesville, VA, USA) and subcloned into pCX4bleo retroviral vector [GenBank: AB086388]. Full-length cDNAs for human *MGMT *and *p53 *were generated by PCR and subcloned into pCX4bleo and pCX4gfp [GenBank: AB296083] retroviral vectors, respectively. Primer sequences used in this experiment included 5'-ATG GAC AAG GAT TGT GAA-3' and 5'-TCA GTT TCG GCC AGC AGG-3' for human *MGMT *and 5'-CTG AAT TCA TGG AGG AGC CGC AGT CAG-3' and 5'-CCG AAT TCA GTC TGA GTC AGG CCC TTC-3' for *human p53*. Other retroviral vectors and the procedure of retroviral-mediated gene transfer were described previously [14]. The murine *EcoVR *was first introduced into NHA cells by using amphotropic virus, in order to make human cells susceptible to the subsequent infection with ecotropic viral vectors. Infected cell populations were selected in blasticidin S (20 μg/ml), G418 (1000 μg/ml), puromycin (500 ng/ml), or zeocine (500 μg/ml) for two weeks. In all cases, cultures arose from polyclonal expansion of infected cells.

### RT-PCR

Total RNA was isolated with the TRI Reagent (Sigma) and reverse transcribed into cDNA using the oligo-dT primer (Invitrogen, Carlsbad, CA, USA) and the Superscript II (Invitrogen). The levels of *MGMT *were analyzed by PCR with the *KOD plus *DNA polymerase (Toyobo, Tokyo, Japan) using the primers described above. PCR primers for *Glyceraldehydes-3-phosphate dehydrogenase (GAPDH) *were described previously [15].

### Soft-agar colony formation assay and xenograft propagation

Soft-agar colony formation assay [15] and xenograft propagation [36] were carried out as described. Female athymic nude mice (BALB/cAJcl-*nu*/*nu*) were purchased from Clea Japan (Tokyo, Japan) and all animal procedures were carried out according to the protocol approved by the institutional Animal Care and Use Committee at Hokkaido University Graduate School of Medicine.

### Histological analysis and immunohistochemistry

Formalin-fixed paraffin-embedded tissues were sectioned and stained with haematoxylin and eosin (H&E) using standard protocols. Immunohistochemistry was performed using anti-Ki-67 (MIB-1; Dako, Glostrup, Denmark) and anti-p53 (DO-7; Dako) monoclonal antibodies.

### Immunoblotting

Protein determination, SDS-PAGE and immunoblotting were carried out as described previously [37], and reactive protein signals were visualized by chemiluminescence using the ECL reagent (Amersham, Piscataway, NJ, USA) or the SuperSignal West Femto reagent (Pierce, Rockford, IL, USA). Antibodies were obtained from the following sources: anti-SV40 large T antigen (Ab-1) and anti-SV 40 small t antigen (Ab-3) monoclonal antibodies (Oncogene Research Product, San Diego, CA, USA); anti-p53 and anti-AKT polyclonal antibodies (Cell Signaling Technology, Beverly, MA, USA); anti-RAS and anti-p27^KIP1 ^monoclonal antibodies (Transduction Laboratories, Lexington, KY, USA); anti-dimethylated Histone-H3 (Me-H3) and anti-acetylated Histone-H3 (Ac-H3) polyclonal antibodies (Upstate); anti-MGMT (MT3.1) and anti-ACTIN monoclonal antibodies (Chemicon International, Temecula, CA, USA); an anti-p21^WAF1 ^monoclonal antibody (Ab-1; Calbiochem, San Diego, CA, USA); an anti-hTERT (L20) polyclonal antibody (Santa Cruz Biotechnology, Santa Cruz, CA, USA).

## Abbreviations

MGMT: O^6^-methylguanine-DNA methyltransferase

GBM: glioblastoma multiforme

NHA: normal human astrocyte

hTERT (T): human telomerase catalytic subunit

H-RasV12 (R): activated H-Ras

myrAKT (A): myristoylated form (active form) of AKT

SV40ER (S): simian virus 40 early region

HDAC: histone deacetylase

VPA: Valproic acid

5-aza-dC: 5-aza-2'-deoxycytosine

RT-PCR: reverse transcriptase polymerase chain reaction

GAPDH: Glyceraldehydes-3-phosphate dehydrogenase

## Competing interests

The author(s) declare that they have no competing interests.

## Authors' contributions

KS designed the research, carried out all experiments except for immunohistochemistry, and drafted the manuscript. TA participated in the design of the study and contributed to new reagents. EA carried out H&E staining and immunohistochemistry. KT participated in epigenetic studies. SK collected and analyzed clinical samples. ST carried out histological analyses, conceived of the study, participated in its design and coordination, and helped to draft the manuscript. All authors read and approved the final manuscript.

## Supplementary Material

Additional file 1 Histopathological analysis of human brain tumors. Formalin-fixed paraffin-embedded tissue sections were stained with H&E. Low- (×100) and high- (×400) magnification images are shown (bar in G1, 200 μm). Tissue sections were also processed for immunohistochemistry using Ki-67 and p53 antibodies. Staining intensities were summarized in Table 2. Click here for file
