# Supplementary figures and images for: O6-methylguanine-DNA methyltransferase is downregulated in transformed astrocyte cells: implications for anti-glioma therapies
Source: Mol Cancer. 2007 Jun 5;6:36. doi: 10.1186/1476-4598-6-36 (PMC1892783; doi:10.1186/1476-4598-6-36)

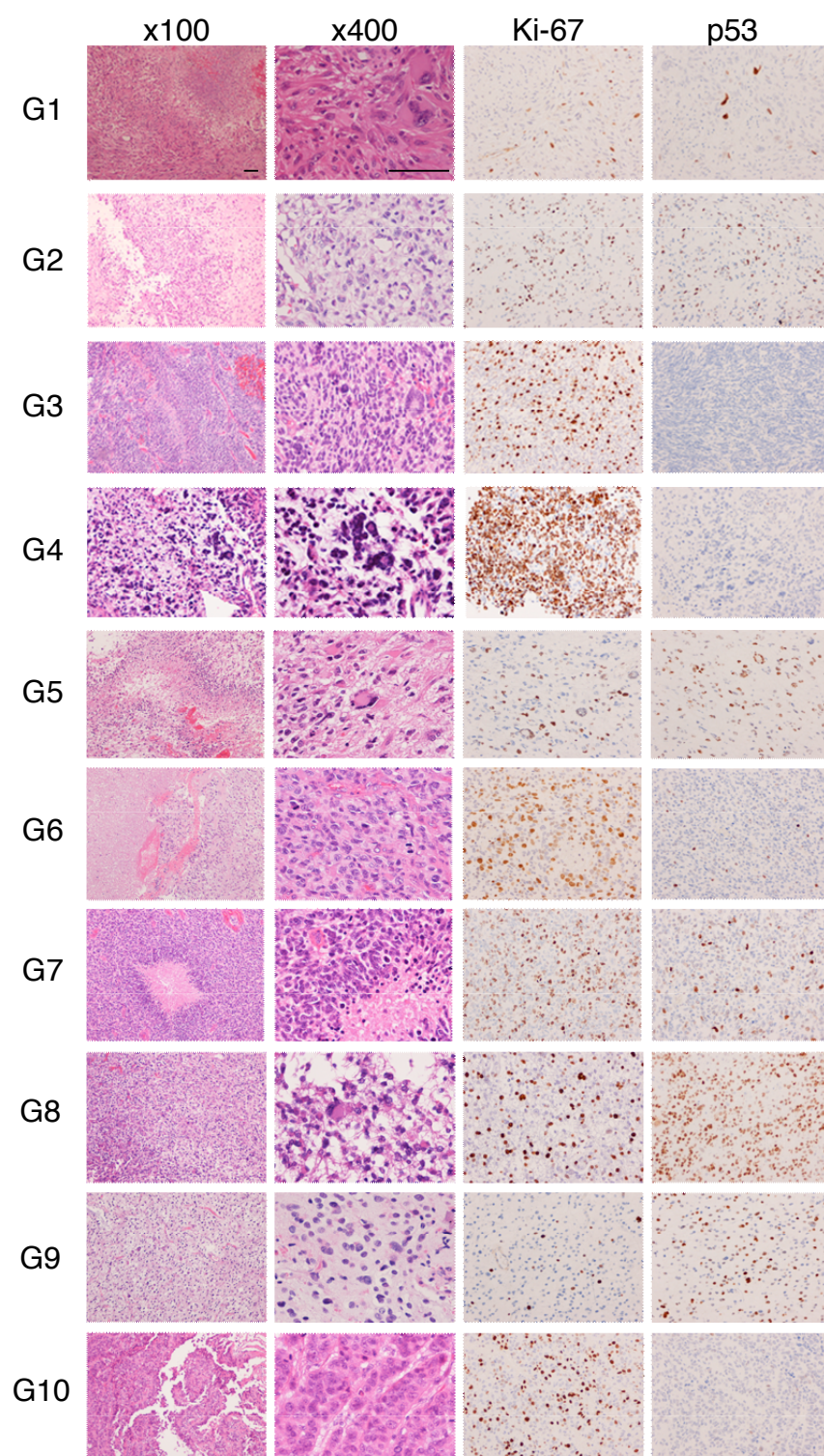

Supplement: Additional file 1 — Histopathological analysis of human brain tumors. Formalin-fixed paraffin-embedded tissue sections were stained with H&E. Low- (×100) and high- (×400) magnification images are shown (bar in G1, 200 μm). Tissue sections were also processed for immunohistochemistry using Ki-67 and p53 antibodies. Staining intensities were summarized in Table 2. [file 1476-4598-6-36-S1.pdf]
